# *Nicotiana glauca* whole-genome investigation for cT-DNA study

**DOI:** 10.1186/s13104-018-3127-x

**Published:** 2018-01-12

**Authors:** Galina Khafizova, Pavel Dobrynin, Dmitrii Polev, Tatiana Matveeva

**Affiliations:** 1 Department of Genetics and Biotechnology, Saint Petersburg State University, Universitetskaya emb. 7/9, Saint Petersburg, 199034 Russia; 2 Research Park, Saint Petersburg State University, 17 Botanicheskaya St, Peterhof, Saint Petersburg, 198504 Russia; 3 Theodosius Dobzhansky Center for Genome Bioinformatics, Saint Petersburg State University, 41A Sredniy Ave, Saint Petersburg, 199004 Russia; 4 National Zoological Park, Smithsonian Conservation Biology Institute, 3001 Connecticut Ave NW, Washington, DC 20008 USA

**Keywords:** *Nicotiana glauca*, Cellular T-DNA, Whole genome sequencing, Genome assembly

## Abstract

**Objective:**

*Nicotiana glauca* (tree tobacco) is a naturally transgenic plant, containing sequences acquired from *Agrobacterium rhizogenes* by horizontal gene transfer. Besides, *N. glauca* contains a wide profile of alkaloids of medical interest.

**Data description:**

We report a high-depth sequencing and de novo assembly of *N. glauca* full genome and analysis of genome elements with bacterial origin. The draft genome assembly is 3.2 Gb, with N50 size of 31.1 kbp. Comparative analysis confirmed the presence of single, previously described gT insertion. No evidence was acquired to support idea of multiple T-DNA insertions in the *N. glauca* genome. Our data is the first comprehensive de novo assembly of tree tobacco and provide valuable information for researches in pharmacological and in phylogenetic fields.

## Objective

*Nicotiana glauca* (tree tobacco) is a member of the *Solanaceae* family, which includes important crops (potato, tomato, eggplant, pepper) and many medicinal plants [1]. This diploid plant is native to South America and is one of the first *Nicotiana* species with *Agrobacterium* cellular T-DNA (cT-DNA) [2]. Its cT-DNA is a partial, inverted repeat, called gT [3]. Tree tobacco belongs to the section *Noctiflorae.* Sequencing of the genomes of *N. tomentosiformis* and *N. otophora* (section *Tomentosae*) and *N. tabacum* (section *Nicotiana*) allowed the detection of previously unknown multiple cT-DNAs [4], raising the question whether there are other T-DNA insertions in the *N. glauca*. NGS data can help answer this question. Besides, *N. glauca* contains a profile of alkaloids different from *N. tabacum* [5]. The plant is used for medicinal purposes. Comparative analysis of genomic data of phylogenetically distant tobacco species will provide valuable information on the genetic basis for various traits, especially secondary metabolism. Our data complement the list of species for the comparative genomics of *Nicotiana*, which opens up new opportunities for pharmacological and phylogenetic studies.

## Data description

One plant isolate was sequenced on Illumina HiSeq machine, yielding in total 210 Gb of raw sequence data. De novo assembly resulted in 385116 scaffolds, with N50 and L50 of 31.1 kbp and 27293 respectively. Genome size suggested by K-mer analysis is 2 Gb, while the final size of the assembled genome equaled 3.2 Gb. Comparative analyses of *N. glauca* scaffolds against genome assembly of *N. tabacum* TN90 cultivar strain resulted in 3.2 Gbp of aligned sequences median identity of 88%. T-DNA analysis revealed sequences homologous to agrobacterial genes *orf13a, orf13, orf14, rolC, rolB* and *mis*. The fragment of T-DNA obtained in the assembly is organized in an imperfect inverted repeat. The similarity of the nucleotide sequences, that we found, and sequence of gT, previously described by Suzuki [3] was 99%, while its similarity to *Agrobacterium* T-DNA is 77–89%. Sequences of PCR fragments, amplified from T-DNA/plantDNA junction areas, coincide with known ones (Acs. AB071335, AB071334).

### Methodology

#### Sample collection

Leaf tissue of aseptic plants *N. glauca* was used for DNA extraction, with a modified version of Doyle and Doyle protocol [6], yielding 30 ng/μl of high molecular weight DNA.

#### Library construction

Purified genomic DNA from *N. glauca* was used to construct both pair-end and mate pair libraries in order to generate a high coverage de novo assembly. A pair-end library with an insert size of 350 bp was constructed using the TruSeq^®^ Nano DNA Library Prep Reference Guide. To improve resolution of repeats during the assembly stage and scaffolding process, one mate pair library with an insert size of 4 kbp was constructed, according to the Nextera^®^ Mate Pair Library Prep Reference Guide.

#### Read sequencing, quality analysis and filtering

Pair-end and mate pair libraries were sequenced on four and two lanes using Illumina HiSeq. Quality of raw reads was analyzed with the FastQC [7] program, followed by filtering and trimming raw PE reads with Trimgalore [8]. Mate pair raw reads were processed and splitted with Nextclip [9] and additionally filtered with Trimgalore [8].

#### Genome assembly

The genome was assembled with the MaSuRCA-3.2.2 genome assembler [10], [config in data file 1].

#### Whole genome alignment of *Nicotiana glauca* and *Nicotiana tabacum*

To identify the location of the *N. glauca* cT-DNA insertion relative to the *N. tabacum* genome, we mapped all *N. glauca* scaffolds to *N. tabacum* scaffolds downloaded from the Sol Genomics Network [11]. To increase accuracy of alignment we masked all known plant repeat classes and their homologs in the *N. glauca* genome. For repeat identification, we used the RepeatMasker software [12] and the latest Repbase Update library from 09.27.2017. For whole genome alignment, we used the Last software [13].

#### T-DNA analysis

The Last software [13] was used to carry out the alignment of the database, containing all known T-DNA-like sequences, that were detected as part of cT-DNA [data file 2], to the *N. glauca* genome. To reaffirm T-DNA/plantDNA junction areas Long PCR was carried out using “LONG PCR enzyme Mix” (Thermo scientific) according to the instructions for the kit (Table 1).

**Table 1:** Overview of data files

Label	Name of data file	File types	Data repository and identifier	License
Supplementary file 1	Methodology description	.docx file	10.6084/m9.figshare.5732427.v1	CC BY
Data file 1	Parameters for the assembly	.txt file	10.6084/m9.figshare.5645854.v1	CC BY
Data file 2	T-DNA database	.fa file	10.6084/m9.figshare.5754120.v1	CC BY

## Limitations

85% of the mate pair library proved to be PCR duplicates, which we filtered before assembling. Low coverage of MP reads resulted in low N50 and big number of contigs and scaffolds. A better quality or/and a bigger number of MP libraries should be used in future to improve the assembly.

## Abbreviations

T-DNA: transferred DNA
PE: pair-end
MP: mate pair

## References

[1] Long N, Ren X, Xiang Z, Wan W, Dong Y (2016). Sequencing and characterization of leaf transcriptomes of six diploid *Nicotiana* species. J Biol Res (Thessalon).

[2] White FF, Garfinkel DJ, Huffman GA, Gordon MP, Nester EW (1983). Sequence homologous to *Agrobacterium rhizogenes* TDNA in the genomes of uninfected plants. Nature.

[3] Suzuki K, Ichiro Y, Nobukazu T (2002). Tobacco plants were transformed by *Agrobacterium rhizogenes* infection during their evolution. Plant J.

[4] Chen K, Dorlhac de Borne F, Szegedi E, Otten L (2014). Deep sequencing of the ancestral tobacco species *Nicotiana tomentosiformis* reveals multiple T-DNA inserts and a complex evolutionary history of natural transformation in the genus *Nicotiana*. Plant J.

[5] Saitoh F, Kawasima N (1985). The alkaloid contents of sixty *Nicotiana* species. Phytochemistry.

[6] Doyle JJ, Doyle JL (1987). A rapid DNA isolation procedure for small quantities of fresh leaf tissue. Phytochem Bull.

[7] FastQC program. https://www.bioinformatics.babraham.ac.uk/projects/fastqc/. Accessed 12 Jan 2017.

[8] Krueger F. Trim Galore!: a wrapper tool around Cutadapt and FastQC to consistently apply quality and adapter trimming to FastQ files. 2015. http://www.bioinformatics.babraham.ac.uk/projects/trim_galore/. Accessed 12 Jan 2017.

[9] Leggett RM, Clavijo BJ, Clissold L, Clark MD, Caccamo M (2013). NextClip: an analysis and read preparation tool for Nextera Long Mate Pair libraries. Bioinformatics.

[10] Zimin AV, Puiu D, Luo MC, Zhu T, Koren S, Marçais G, Yorke JA, Dvořák J, Salzberg SL (2017). Hybrid assembly of the large and highly repetitive genome of *Aegilops tauschii*, a progenitor of bread wheat, with the MaSuRCA mega-reads algorithm. Genome Res.

[11] Sol Genomic Network. https://solgenomics.net. Accessed 25 Feb 2017.

[12] Tarailo-Graovac M, Chen N (2009). Using RepeatMasker to identify repetitive elements in genomic sequences. Curr Protoc Bioinform.

[13] Kiełbasa SM, Wan R, Sato K, Horton P, Frith MC (2011). Adaptive seeds tame genomic sequence comparison. Genome Res.

